# An Efficient Approach for Web Indexing of Big Data through Hyperlinks in Web Crawling

**DOI:** 10.1155/2015/739286

**Published:** 2015-06-07

**Authors:** R. Suganya Devi, D. Manjula, R. K. Siddharth

**Affiliations:** Department of Computer Science and Engineering, College of Engineering, Guindy, Anna University, Chennai 600025, India

## Abstract

Web Crawling has acquired tremendous significance in recent times and it is aptly associated with the substantial development of the World Wide Web. Web Search Engines face new challenges due to the availability of vast amounts of web documents, thus making the retrieved results less applicable to the analysers. However, recently, Web Crawling solely focuses on obtaining the links of the corresponding documents. Today, there exist various algorithms and software which are used to crawl links from the web which has to be further processed for future use, thereby increasing the overload of the analyser. This paper concentrates on crawling the links and retrieving all information associated with them to facilitate easy processing for other uses. In this paper, firstly the links are crawled from the specified uniform resource locator (URL) using a modified version of Depth First Search Algorithm which allows for complete hierarchical scanning of corresponding web links. The links are then accessed via the source code and its metadata such as title, keywords, and description are extracted. This content is very essential for any type of analyser work to be carried on the Big Data obtained as a result of Web Crawling.

## 1. Introduction

In today's fast-paced world, technology has vastly improved and has made an impact in every aspect of a human life. This has resulted in large accumulation of information across all individuals and fields resulting in the presence of Big Data. Big Data processing is an integral part of every individual's daily life aiming at dealing with billions of users' interactive data. This gives rise to the need for real-time Big Data processing to be integrated with the application systems. The widespread use of Internet provides a good environment for data inspection. Big Data processing and analysis poses several issues and challenges as a result of the accumulating quantity of information. It becomes an important aspect to handle all the information and present it in a way as required.

This paper handles one section of the Big Data presence and application and brings forward an optimal and efficient method to extract and process it simultaneously. There exists thousands of links associated with each URL linked with the Internet. This paper first focuses on identifying the best method to crawl these links from the corresponding web URLs. It then builds an efficient extraction to identify the metadata from every associated link. This would give rise to the accumulation of the documents along with the corresponding title, keywords, and description. This information can be used in the future to classify the Big Data documents effectively as required by the application.

The aim of this paper is to propose an efficient method to crawl and index the links associated with the specified URLs. As the World Wide Web is a vast area, thousands of links are scattered across the Internet across all the websites. Using the concept of hyperlinking, the documents are crawled from the websites and the source code is accessed to extract the metadata. It is essential to identify this information so as to integrate it with any application and analyze the Big Data thereafter.

## 2. Literature Review 

Due to the tremendous advancement in the information available on the World Wide Web, it has become rather inevitable to employ the use of tools to identify the information resources and to process and analyze them. This paved the way for server-client-side systems essential for efficient knowledge mining [1]. Cooley et al. present a needful description of web mining which forms the crux of Web Crawling. They were the foremost in establishing a general architecture for a system to carry out the usage of web mining. However the technology and needs have vastly changed since then and have developed the need for more advanced methods. Still the taxonomy proposed by them in regard to web mining has been carried on till date to form the basis of Web Crawling.

The rapid development of Internet and cloud computing in the recent years has paved the way for the tremendous growth in every field of business and industries. “Big Data represents the information assets characterized by High Volume, Velocity and Variety to specific Technology and Analytical Methods for its Transformation into Value [2]. The quantity of data that is generated is very important in this context. It is the size of the data which determines the value and potential of the data under consideration and whether it can actually be considered Big Data or not. The name ‘Big Data' itself contains a term which is related to size and hence the characteristic.” Big Data has been speeding up its development to trend as the most important topic that attracts considerable attention from researchers, academicians, industries, and governments all around the globe.

Jin et al. [3] present briefly the concept of Big Data combined with its features and challenges. They put forward a few necessary conditions for the success of a Big Data project. Since Big Data consists of a large amount of information, it is necessary to identify the specific requirements regardless of its nature. Also it is said that the kernel data/structure is to be explored efficiently. The most important point noted by them is the application of a top-down approach to handle Big Data. This allows the isolated solutions to be put together to come to a complete solution. They also support the conclusion of a project by integrating the solution.

Gandomi and Haider [4] present the various analytics methods used for Big Data. Since a majority of the available Big Data is unstructured, their focus on this dataset provides a wider understanding on the applications on Big Data. They have stressed the importance of real-time analytics which are bound to become the major field of research in the future. This is highly due to the growth of social networking and mobile apps. They have identified that predictive analysis has been dominating all fields of analytics and present the case for new methods to address the differences of Big Data.

Jagadish [5] has diffused the various abnormalities of Big Data by analyzing a few common myths associated with Big Data and exposing the underlying truth behind them. The most important myth which is busted is that data reuse is low hanging fruit. He further goes on to specify that the reuse of data is critical to handle and promises great future.

Singh and Reddy [6] presented an in-depth analysis of different platforms available for performing Big Data analytics. They provide a detailed analysis on the various advantages and disadvantages possessed by the data processing platforms. This aids researchers in identifying the right platform based on the available Big Data and computational requirements.

Najafabadi et al. [7] explore the important problems in Big Data analytics and how deep learning can be employed for addressing these issues. They further provide information as to why deep learning has an advantage over conventional machine learning algorithms. They specify how deep learning can be used to automatically extract complex data interpretations from large volumes of unstructured data.

Web mining refers to the branch of data mining that works with the analysis of World Wide Web and is found to originate from data mining, World Wide Web, and Internet Technology. Lately though, Semantic Web has made a drastic impact on the concept of web mining [8]. Berendt et al. proposed the idea of Semantic Web which aims at not just accessing the information but also putting forward its usage. This gives rise to the need for Web Crawling and identifying the areas and their specific requirements.

Web mining also is further classified into Web Content Mining [9], Web Structure Mining [10], and Web Usage Mining [11].

David et al. [10] proposed a method to structurally crawl the web. They identified that though the amount of data crawled is tremendous, there exists no reason for the crawled data to be disorderly. They focused on analyzing the behavior of the user using a mathematical technique and later identified the themes of hyperlinked communities based on the above findings. This method facilitated the way of specific Web Crawling based on the user's conduct.

Kosala and Blockeel [11] further simplified the concept of web mining by classifying it into categories, thus making it easier to identify the field of Web Crawling for future works. However they had left the concept of information integration hanging by a thread. This serves an important purpose in recent times as integrated information is more effective than unprocessed data.

The search engine technology is the sole reason for the development of the World Wide Web. Search engines act as the main doorway for the access of information from the World Wide Web. The system proposed by Singh et al. in [12] benefits businesses by providing them with the ability to locate information of a common interest amidst the huge content available in the Internet. However this system fails to satisfy the property of information integration which has risen up to be an important factor in today's information retrieval technology.

The System mentioned by Pandey and Olston in [13] populates an indexed database of web documents which is used by search engines to respond to the queries as specified by the analyser. This system though not only fails at information integration but also provides no methods to update and integrate new links with the previously crawled ones.

The two main types of crawling are generic and focused crawling. Generic crawlers [14] are used to crawl documents and links of varied topics while focused crawlers [15] limit the number of retrievals based on some prior obtained knowledge. However Arasu et al. have specifically mentioned in [14] that the usage of generic crawlers is more essential in a real-life application as the focus on crawls is bound to vary from user to user with respect to their changing prior knowledge.

Another important question that arises from Web Crawling is the type of query that is to be crawled by the system. Ramalingam and Manjula have surveyed the different kinds of queries in [16] based on their purpose and this paves the way for identifying what queries are to be crawled for what purposes. In their paper, they had pointed out that durable queries are the most efficient ones as they are expected to satisfy most of the needs of the user. And for this purpose this paper focuses on crawling queries that hold durability over a span of time.

Web Crawlers are used to build the repositories of web documents so as to index the retrieved information and further analyse them [17]. Web Crawling issues such as efficient resource usage have been dealt with previously [18–20].

## 3. System Architecture

The system proposed in this paper uses the algorithmic process for Web Crawling as in [21]. The most effective way to crawl a web is to access the pages in a depth first manner. This allows the crawled links to be acquired in a sequential hyperlink manner. Kumar et al. have proposed a parallel Depth First Search Algorithm in [21] which paves the way for the system in this paper. Depth First Search Algorithm allows the system to reach the hyperlinks associated with one page before moving on to the next page. Also this system is developed on the backdrop of the Google idea in [22]. However the system takes a step forward by incorporating information integration along with Web Crawling so as to obtain processed data as required by most researchers. This process involves a concurrent working of Web Crawling and metatag extractor efficiently. The system uses the concept of metatag extraction to store the URL, title, keywords, and description in the database. This information can be obtained from the HTML content of every web page. In the future, it can be processed to store the body of the document after converting it to plain text if required by the application.

The features of the proposed system are as follows.

- Identify and index the web through hyperlinks.
- Access new pages in the old sites through the hyperlinks.
- Store the metatag content in database for future use.
- Avoid calculating PageRank as it is time-consuming.
- Alter robots to control the contents the system can access.

**Figure 1:**
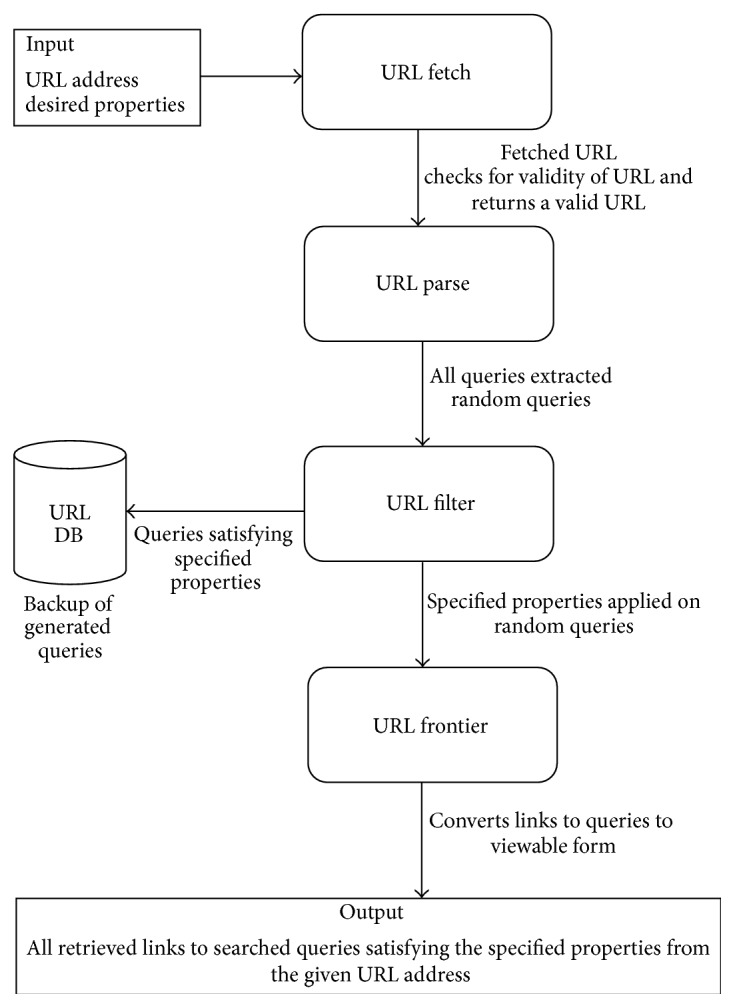
Block diagram of Web Crawling system.

The entire Web Crawling system is depicted in the Block Diagram as shown in Figure 1. The block diagram consists of the process by which the documents are crawled from a web URL as specified by the analyser. The system proposed in this paper makes sure that it lies within the norms of Web Crawling Ethics.

The important point to be noted here is that this paper deals with Big Data. Hundreds of thousands of documents are to be crawled and the process is expected to continue without any interruption. As a result, the metatag extraction is to be designed in such a way so as to accommodate the continuous crawling of Big Data. The system proposed in this paper keeps track of the web documents crawled and simultaneously updates the metatag extraction, thereby avoiding overlap as well as duplicate records.

The crawler allows the analyzer to specify the link(s) from where the hyperlinks are to be crawled. This, along with certain desired properties like specifying the link depth, is to be provided by the analyzer. The next steps involve the verification of the specified URL which is carried out with the help of a URL API validator. This is followed by accessing all the hyperlinks associated with the specified URL. On reaching the condition of the specified properties, the crawler either identifies a different path or terminates. The entire process backs up the generated queries for further research purposes. These links are then stored in the database along with their corresponding metadata extracted as specified above.

## 4. Building the System

The system proposed in this paper functions as a search bot to crawl the web contents from a site. The system is built by developing the front end on  .NET framework on Visual Studio 2012 supported by the Microsoft SQL Server Compact 3.5 as the back-end database. It is then used to interpret the crawled contents based on a user created file named robots.txt file. The working of this system is based on ability of the system to read the web URL and then access all the other pages associated with the specified URL through hyperlinking. This allows the user to build a searchable indexer. This is facilitated by allowing the system to access the root page and all its subpages. The robots.txt file can be used to control the search engine, thereby allowing or disallowing the crawling of certain web pages from the specified URL.

The crawling algorithm implemented by Google in its search pages forms the foundation of the crawler presented in this paper (Algorithm 1). Before the crawler could access the hyperlinks, it creates a robots text file which stores information on where the crawler could access and where it cannot. This information can be accessed from the HTML content of the page. The HTML content consists of a metatag which specifies the position of indexing and following associated with that corresponding page. The metatag is named robots and its contents specify index/noindex and follow/nofollow which can be used to identify whether the link can be indexed or followed. The ability to override these robots contents lies with most antivirus algorithms.

It is important to specify the details of the client application and information about the crawler in the HTTP header. This facilitates to the analyzer understanding the compatibility details of the crawler. It is also specified that this crawler uses HttpWebRequest and HttpWebResponse for downloading the files as it allows the facility to set downloading time-out.

## 5. Software Implementation and Description

A local database is created in the Microsoft SQL Server Compact Edition 3.5 named SearchbotData and the corresponding dataset named SearchbotDataSet is added.

The table with the records as shown in Table 1 is added to the created database. This table will be used to store the results of the system.

A collection of string is then declared to store the links which wait for indexing. An important concept to be noted in Web Crawling is that there exist billions of pages in the World Wide Web and this number keeps increasing every day. As a result there cannot be a system which would finish indexing at all. The system proposed in this paper makes sure the system stops by specifying a variable which is used to check before indexing the next page. This is carried out by declaring a Boolean variable. The crawler would verify this variable before moving on to the next hyperlink for indexing.

The main function of the crawler is the scanning of the hyperlinks. There should exist at least one link in the waiting collection for the hyperlinks to be indexed and the algorithm to begin crawling. The main idea of this paper is to avoid duplicates and provide an efficient method to crawl all the links of the specified URL. As a result the entire scanning process is made available inside a loop along with the abovementioned variable. The basic idea of this scanning method is as follows.

- There should exist at least one page with hyperlinks.
- After parsing the page, it is deleted from waiting.
- Identified hyperlinks are added to the waiting queue.
- Parse the pages in the hyperlinks and repeat the process.

It is noted that this scan process is maintained in another thread so as to not interfere with the metatag extraction module.

This process results in accessing all the web pages associated with the specified URL. It is now necessary to extract the metatag content from the retrieved web pages. The most simple and efficient way is to parse the HTML elements using the usage of regular expressions. Regular expressions represent a sequence of characters that forms a pattern for string searching or string matching. It should be noted that the regular expressions are considered to be the most powerful tools for string comparison.

Gouveia et al. in [23] have specified the usage of regular expressions in string comparisons. The proposal in that paper is used to match the metatag content in the HTML page as per the requirements of the analyser. The following regular expression is used for identifying and parsing the metatags:

 <meta(?:∖s+([a-zA-Z_∖-]+)∖s∗∖=∖s∗([a-zA-Z_∖-]+|∖"[^∧^∖"]∗∖"))∗∖s∗∖/?>

Parts of string matching can be stored using these regular expressions, which stores the names and values of the required attributes. The abovementioned regular expression extracts all the contents of the HTML page irrespective of its characters. This process is passed through a switch file to separate the contents of the metatag as specified in Table 1. The entire content is scanned to identify the title, keywords, and description and the following regular language is then updated in Table 1. In case of the absence of certain information, the entry in the table can be left empty.

This process will identify the title, keywords, and description of the base page as well as the hyperlinks. Once the content is identified, it is then parsed and then added to the results table.

## 6. Crawling Algorithm

The most efficient algorithm to be used and is used for Web Crawling is the Depth First Search Algorithm. This is a powerful algorithm which is used to travel through the search by starting at the base page and traversing deeper through every found hyperlink page. It then backtracks and moves to the adjoining hyperlink pages [24].

This is the most useful search with reference to accessing the deep depths of the cyberspace. Here backtracking is the most important concept and is used to identify the next unvisited link and this process is repeated in a similar fashion to all hyperlinks. The crawling algorithm accesses the leftmost child of the current vertex, if one or more children exist. It then traverses in the same direction until there are no more children in that path. During this traversal, the links passed by the crawler are stored in a stack which would be used for backtracking. This allows the analyzer to safely assume that all the links are visited at least once during the entire traversal.

It can be noted that the Depth First Search Algorithm is implemented with the help of a stack imbibing the concept of LIFO (Last in First Out). The greatest hardship lies in not allowing the crawler to wander off into the depths of cyberspace and efficiently trace through all the hyperlinks. This is brought about by keeping track of the link depth, thereby allowing us to stay in track with the depths of the links in cyberspace. Link depth refers to the number of “clicks” a page is away from the root link specified, where a “click” denotes following a link on a page.

The algorithm used for crawling in this paper will be explained in detail in the following. Firstly, the vertex from where the search should begin is determined. This is provided by the user or analyzer as the root link or URL. The link specified in the crawler acts as the root, thereby automatically assigning it to be the vertex of the search.

The crawler also provides the opportunity to specify the maximum link depth that the crawler can access in case of narrowing down the search for specific research purposes. This is followed by verifying if the vertex specified by the analyzer is the same as the goal state as required. This comparison acts as the condition for the termination of the loop. When the vertex link is identified to be equivalent to the goal state, the crawler searches for other possible directions it could reach from this. In case of absence of any other link route, the algorithm terminates, thereby presenting the analyzer with the required results within the specified conditions.

In the event of specifying a link depth, the crawler undergoes another comparison condition to check if the current vertex is equivalent to the vertex in the link depth specified. This is made to ensure that the crawler stays within the specified conditions and does not move beyond the specified boundary.

The crawling algorithm begins the mechanism only when the abovementioned conditions are not satisfied; that is, the root vertex should not be equivalent to the goal state, and, if so, it should possess some untraversed direction from the root vertex. Also the current vertex should not exceed the link depth specified.

When the current vertex reaches the state of the specified link depth, the crawler is developed with the ability to backtrack its path. This provides the crawler with the opportunity to traverse all possible hyperlinks within the specified boundaries.

The backtracking is done with the help of a stack. As the vertex moves from the root to the goal state, the expansion of the hyperlinks is stored in a stack. When reaching the specified boundaries, the crawling algorithm is called recursively for all the vertices of the stack. This process is repeated for every link reached by the crawler, thereby allowing it to keep track and access the hyperlinks as specified.

## 7. Results and Discussion

In this section the results of the proposed system are discussed. The system is designed to eliminate all unnecessary information from the user and analyser. The system takes as input only the specified URL from the user. The system is developed in such a way so as to cycle the loop over a period of ten seconds. This allows the bot time to refresh and access new pages, thereby eliminating the overlap and duplicate pages.

The overall performance of the system is calculated using precision, recall, and *F*-measure.

Recall rate is calculated by using the following equation:

(1) $$\begin{array}{c} \text{Recall}=\frac{\text{Number}\;\;\text{of}\;\;\text{relevant}\;\;\text{pages}\;\;\text{retrieved}}{\text{Total}\;\;\text{number}\;\;\text{of}\;\;\text{relevant}\;\;\text{pages}}. \end{array}$$

Precision is calculated by using the following equation:

(2) $$\begin{array}{c} \text{Precision}=\frac{\text{Number}\;\;\text{of}\;\;\text{relevant}\;\;\text{pages}\;\;\text{retrieved}}{\text{Total}\;\;\text{number}\;\;\text{of}\;\;\text{retrieved}\;\;\text{pages}}. \end{array}$$

*F*-score is calculated by using the following equation:

(3) $$\begin{array}{c} F\text{-score}=2\cdot \frac{\text{Precision}\cdot \text{Recall}}{\text{Precision}+\text{Recall}}. \end{array}$$

**Figure 2:**
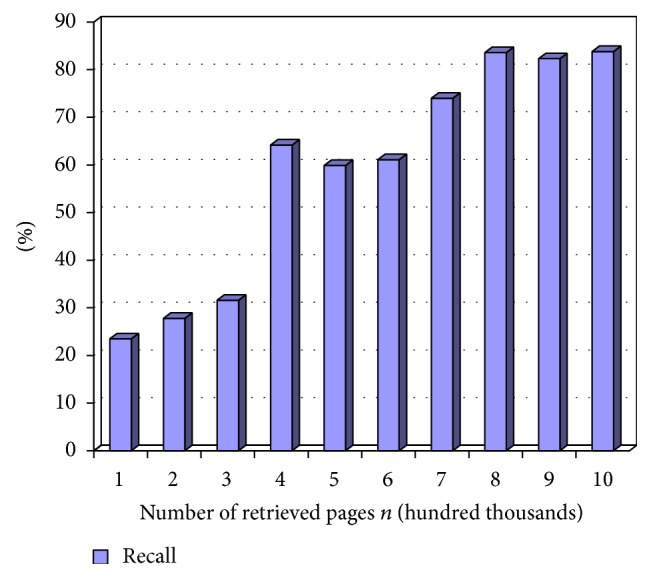
Recall values at *n*.

**Figure 3:**
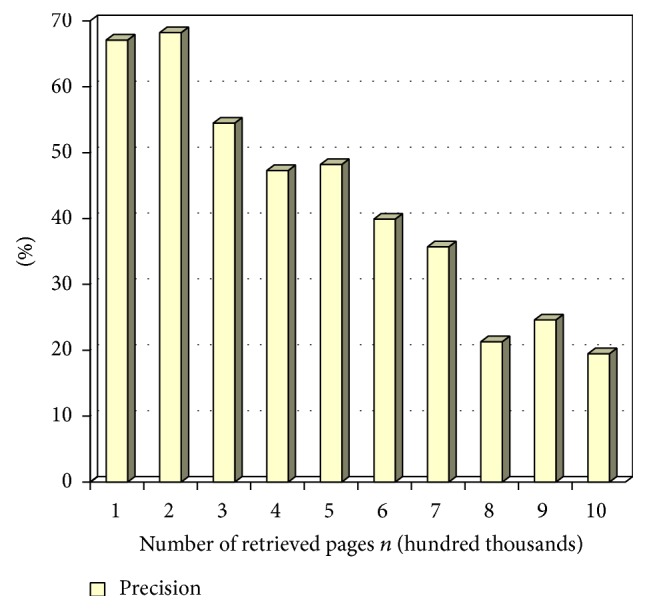
Precision values at *n*.

**Figure 4:**
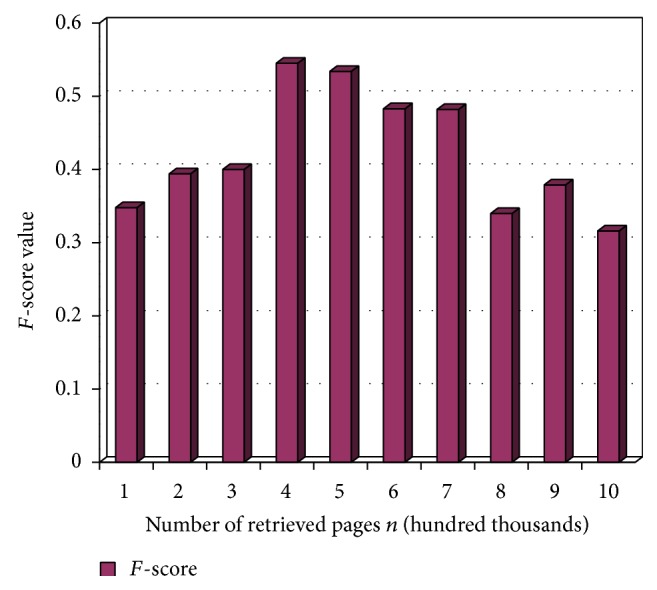
*F*-score values at *n*.

Figures 2, 3, and 4 show precision, recall, and *F*-score measure on retrieved pages at different time intervals in terms of percentage. The term *n* represents the total number of links crawled by the system. This variable is represented in the *x*-axis of the tables in the following figures while the *y*-axis is represented by the percentage value. The scale of the *x*-axis is represented at 100000 pages for every unit. The experiment is carried out on 1000000 links extracted using the system.

Recall is measured for durable relevant pages from the large World Wide Web collection.  Figure 2 shows the recall measure by varying the number of retrieved pages, *n*. Recall is calculated by dividing the number of relevant pages retrieved by total number of relevant pages. The value obtained is converted to percentage and represented in the chart in Figure 2.

Precision is measured for durable relevant pages from the large World Wide Web collection. In Figure 3 precision is measured by varying the number of retrieved pages, *n*. Precision is calculated by dividing the number of relevant pages retrieved by total number of retrieved pages. The value obtained is converted to percentage and represented in the chart in Figure 3.

*F*-score is measured for durable relevant pages from the large World Wide Web collection. In Figure 4, *F*-score is measured by varying the number of retrieved pages, *n*. *F*-score is calculated by doubling the product of precision and recall and dividing it by the sum of precision and recall. The value obtained is converted to percentage and represented in the chart in Figure 4.

Table 2 shows the precision, recall, and *F*-score for different values of *n*.

**Figure 5:**
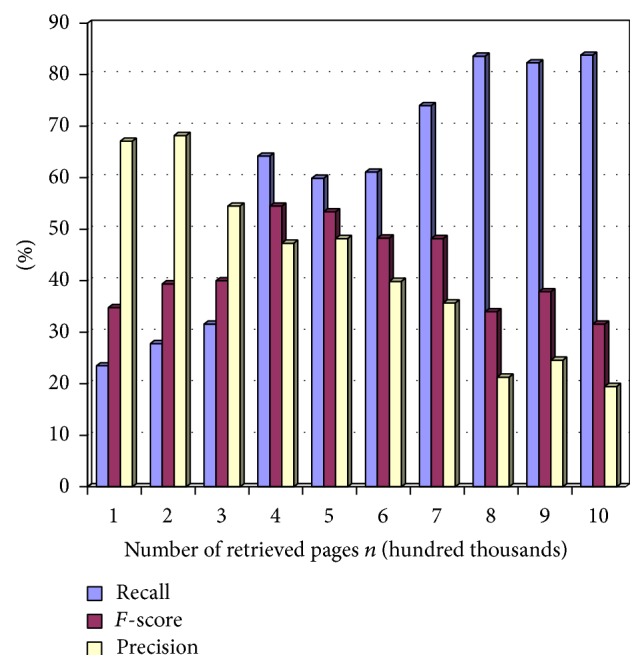
Performance measure.

Figure 5 shows the performance evaluation for the above three measures in percentage.

Minhas and Kumar have recently proposed a modification on a Tropical Web Crawler [25]. In that paper, they had implemented a domain specific focused crawler. The experiment in this paper is also domain specific as it is very useful in saving time as well as resources. They had specifically compared their LSI based crawler with breadth first crawler as well as keyword based crawler and have made it clear that the performance of their crawler is the most superior among them all. This study has been one of the most recent conclusions in identifying the most efficient crawler. As a result, the experiment in this paper is also put through the same recall and precision analysis, to devise a comparative analysis between the two crawlers and establish the results.

Table 3 shows the recall, precision, and *F*-score values comparison between the abovementioned two crawlers.

**Figure 6:**
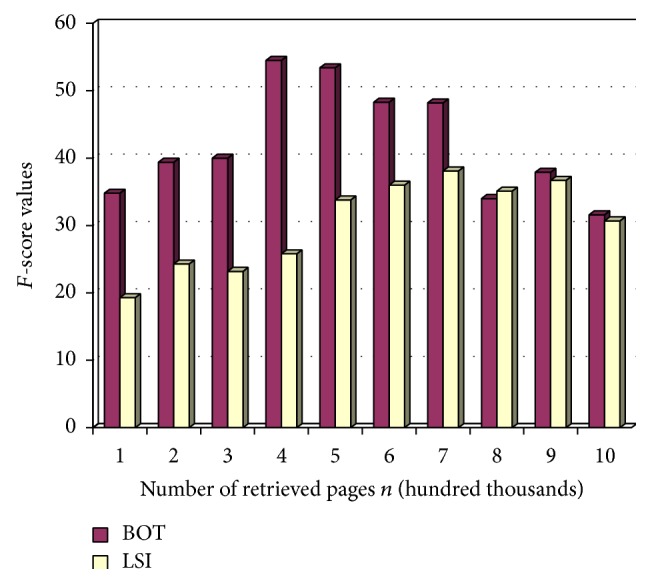
*F*-score value comparison.

Figure 6 shows the most important *F*-score value comparison between the two crawlers.

From the *F*-score values compared between the two crawlers, it can be clearly seen that the BOT crawler vastly outnumbers the LSI crawler implemented by Minhas and Kumar [25]. As the LSI crawler had recently been proved effective, the BOT crawler specified in this paper surpasses the effective values of the LSI crawler, thus making this the most efficient crawler for Big Data in recent times.

## 8. Conclusion

Based on the above findings, it can be concluded that the system progresses as the number of crawled pages increases. After an initial boost, it is found that the durable pages that are required to be crawled occur with more probability as the total number of pages increases. This shows that when applied to a real-time application which handles millions of data, the performance numbers are bound to reach the maximum efficiency, thereby presenting the most efficient Web Crawling System. The advantageous addition to this system is its information integration with the simultaneous metatag extraction.

The proposed system is mainly focused on building a database of pages and links from the World Wide Web. It also focuses on recrawling frequently changing web pages so as to keep the contents of the database current. Also the integration of a metatag extractor within the crawling leads to profound possibilities based on the requirements of the analyser. This greatly reduces the need to implement separate extraction modules in the projects.

Future works could be done on reducing the amount of bandwidth required to generate this system and make it accessible to the next level of links.

## Figures and Tables

**Algorithm 1:**
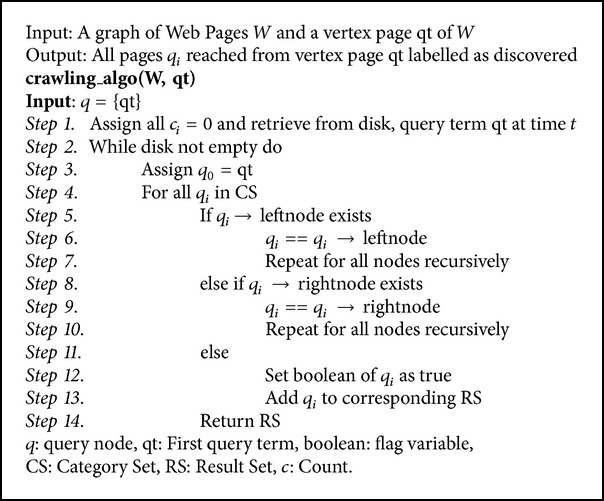


**Table 1:** Table results.

Column name	Data type	Length	Allow nulls	Primary key	Identity
id_result	int	4	No	Yes	Yes
URL_result	nvarchar	500	No	No	No
title_result	nvarchar	100	Yes	No	No
keywords_result	nvarchar	500	Yes	No	No
description_result	nvarchar	1000	Yes	No	No

**Table 2:** Recall, precision, and *F*-score values at *n*.

*n*	100,000	200,000	300,000	400,000	500,000	600,000	700,000	800,000	900,000	1,000,000
Precision (*P*)	0.671	0.682	0.545	0.473	0.482	0.399	0.357	0.213	0.246	0.195
Recall (*R*)	0.235	0.278	0.316	0.642	0.599	0.611	0.74	0.836	0.823	0.838
*F*-score	**0.348**	**0.394**	**0.4**	**0.545**	**0.534**	**0.483**	**0.482**	**0.34**	**0.379**	**0.316**

**Table 3:** Recall, precision, and *F*-score value comparison.

*n*	200,000		400,000		600,000		800,000		1,000,000	
Crawler	LSI	BOT	LSI	BOT	LSI	BOT	LSI	BOT	LSI	BOT
Precision (*P*)	0.405	0.682	0.331	0.473	0.317	0.399	0.246	0.213	0.201	0.195
Recall (*R*)	0.174	0.278	0.212	0.642	0.417	0.611	0.614	0.836	0.647	0.838
*F*-score	**0.243**	**0.394**	**0.258**	**0.545**	**0.36**	**0.483**	**0.351**	**0.34**	**0.307**	**0.316**
